# Bi-layer cBN-based composites reinforced with oxide and non-oxide microfibers of refractory compounds

**DOI:** 10.1038/s41598-024-60856-3

**Published:** 2024-05-02

**Authors:** Serhiy Klymenko, Hao Zhang, Denys Savchenko, Anatoliy Chumak, Sergiy Klymenko, Yuriy Melniychuk, Andriy Manokhin, Valerii V. Kremenytskyi, Victor M. Novichenko

**Affiliations:** 1 V.M. Bakul Institute of Superhard Materials National Academy of Sciences of Ukraine, Kyiv, Ukraine; 2 Scool of Mechanical Engineering, Beijing Institute of Technology, Beijing, China; 3 Technical Center of the National Academy of Sciences of Ukraine, Kyiv, Ukraine

**Keywords:** cBN-based composites, Turning, SiC_w_, Al_2_O_3w_ microfibers, Microstructure and mechanical properties of PcBN, Tool life and wear, Mechanical engineering, Composites

## Abstract

Research into new composites utilizing cubic boron nitride (PcBN) shows promise for enhancing cutting tool performance. The unique properties of these materials stem from the addition of microfibers made of refractory compounds to their structure. This study looks at developing two-layer composites based on cBN group BL, reinforced with SiCw and Al_2_O_3w_ microfibers. The goal is to improve tool stability when cutting hardened steels with impact loads. PcBN composite samples were made by sintering a mixture of cBN powder with bundles and microfibers under 7.7 GPa pressure. Bond material selection was based on analyzing the relationship between Poisson's ratio (η) and plasticity parameter (G/B). The density, Young's modulus, Poisson's ratio, and hardness of the composites were determined, and the microstructure of samples with TiCN bond was studied. Tool-life tests were conducted on two-layer cutting inserts made of PcBN reinforced with SiCw and Al_2_O_3w_ microfibers during the machining of hardened KhVG steel (HRC 55) under impact loads at cutting speeds of 100 and 200 m/min.

## Introduction

The advancement of industry has led to an increased utilization of difficult-to-machine alloys and steels, known for their enhanced mechanical properties. Consequently, cutting tools equipped with composites based on Polycrystalline Cubic Boron Nitride (PcBN) have become widely employed for the mechanical processing of products made from these materials. The operational capabilities of PcBN cutting tools are determined by the composite's structural characteristics and phase composition, related to the content and size of BN grains, as well as the type, composition, and quantity of the binder component^1–3^.

Currently, according to the ISO (International Organization for Standardization) 513–2012 standard, three types of PcBN are recognized for cutting tool applications^4,5^: those with a low content of cBN (BL group) constituting 45–65 vol% of the total volume; those with a high cBN content (BH group) constituting 70–95 vol% of the total volume; those with a protective coating (BC group).

BL group composites are made with a multiphase binder based on ceramic compounds—Titanium Nitride (TiN), Titanium Carbonitride (Ti(C, N)), Titanium Carbide (TiC), Tantalum Nitride (TaN), Titanium Diboride (TiB_2_), Silicon Nitride (Si_3_N_4_), Silicon Carbide Whiskers microfibers (SiC_w_), a family of ternary carbides and nitrides (MAX phases).

Employing a ceramic binder enhances the cutting tools wear resistance, which occurs due to chemical interactions between cBN and the material being processed, PcBN oxidation, as well as the diffusion of boron and nitrogen from the composition of cBN to the processed material. This is particularly vital during continuous processing at high cutting speeds^6^.

BH group composites have higher impact toughness than BL group PcBN and are most often made using a metal binder. Tools crafted from such materials are effective in both the continuous processing of hardened steels and machining under shock loads^7^.

Applying protective coatings to the working parts of cutting tools with BC group composites increases their reliability during the initial run-in phase as well as when processing abrasive steels and alloys under dynamic loads^8^.

Works^7,9^ has explored the continuous, semi-continuous, and intermittent turning of hardened steel using cutting tools equipped with PcBN of the BH group. Findings indicate that these cutting tools exhibit longer service life when processing discontinuous surfaces compared to continuous ones.

The authors of the paper^10^ investigated three types of cutting tool’s performance with working parts made from PcBN during continuous, semi-continuous, and intermittent machining of hardened AISI 4340 steel (52 HRC). The study focused on composites with a 50 vol% cBN content and a TiCN binding phase, and those with a 50 vol% cBN content and a TiC binding phase. Such cutting tools are used for the continuous turning of products made from hardened steels, superalloys, abrasive materials, and products obtained by powder metallurgy methods. Cutting tools with a cBN content of 65% and a TiCN binding phase are used for processing products made of hardened steels, primarily under intermittent cutting conditions of medium and significant intensity. Lastly, cutting tools with a cBN content of 90 vol% and a Co–Ni binding phase are utilized for processing products made of gray and high-strength cast iron, as well as steel products obtained by powder metallurgy methods. Continuous processing of steel exposes the cBN grains in the working part of the cutting tool to thermal and chemical stresses, which in turn leads to a higher wear rate of the cutting tool, especially noticeable at high cutting speeds TiC and TiCN binders, used in composites with cBN contents of 50 vol% and 65 vol%, respectively, contribute to slower cutting tool wear under continuous cutting conditions. The cutting tool equipped with a composite containing 50% cBN and the highest volume percentage of corrosion-resistant material performs best under these conditions. It has been determined that during intermittent processing, the wear intensity of BN grains in the composite due to chemical interactions in the cutting zone decreases. However, the wear of the binder component occurs at an increased rate. Under such conditions, cutting tools equipped with a composite containing the highest cBN content exhibit the longest tool life.

Research^11^ has indicated that despite the high oxidation resistance of tantalum nitride in PcBN composites with a TaN binder, facilitated by the formation of a tantalum oxynitride film—such composites fail to achieve the desired outcomes. This shortfall is attributed to the significant difference in the coefficient of thermal expansion (CTE) and the modulus of elasticity among the composite's constituents. Further analysis of the elastic properties of the produced PcBN reveals that TaN composites exhibit a substantial contribution from ion-metal bonds, which is considered undesirable due to the potential for chemical interactions with iron-based alloys.

Enhancing the tool life of PcBN cutting tools in the BL group during intermittent processing can be achieved by altering the chemical composition and creating a specific structure within the composite.

In^11^ incorporating silicon carbide (SiC_w_) and aluminum oxide (Al_2_O_3w_) fibers of sizes ranging from 0.5–2.0 μm and 15–50 μm, respectively, has been proposed as a means to modify the composite structure. Al and TaN compounds were utilized as matrix materials.

Considering the differences in the thermal expansion coefficients of the components^11^, it has been suggested to substitute the bonding material based on the TiC, TiN, Ti(C,N) systems with additives of Al compounds, for a composition based on NbN and to reinforce the composite structure with Al_2_O_3w_ and Si_3_N_4w_ fibers. This modification demonstrated that materials reinforced with Si_3_N_4w_ fibers, comprising 10 vol%, exhibit the optimal combination of mechanical properties, including hardness, Young's modulus, and fracture toughness. Nevertheless, these materials were deemed unsuitable for cutting tools during intermittent machining due to their low wear resistance. In contrast and despite relatively lower crack resistance and structural heterogeneity, composites with Al_2_O_3w_ microfibers showcased the best wear resistance among cutting tools tested. The authors attribute the superior performance to the higher chemical and diffusion stability of Al_2_O_3w_ compared to Si_3_N_4w_.

In study^12^, SiC_w_ fibers with a purity of 90% were utilized, boasting an average fiber diameter ranging from 0.1 to 3.0 μm. The composite was interspersed with SiC_w_ microfibers at volumetric proportions of 5 wt%, 10 wt%, 15 wt%, and 20 wt%. The researchers observed that incorporating up to 5 wt% of SiC_w_ microfibers enhances the composite's density. Additionally, increasing the microfiber content to 10 wt% resulted in a maximum bending strength of 695.85 MPa, a 23.5% improvement over the base PcBN. This enhancement in bending strength is attributed to the reinforcing effect of SiC_w_ microfibers at the grain boundaries, enhancing the composite's load-bearing capacity and resistance to microcrack growth. The highest fracture toughness value was achieved for the cBN-based composite with a mass content of SiC_w_ fibers of 15%, and it is 7.38 MPa·m^1/2^, which is 41.9% higher than that of the unreinforced composite.

The underlying mechanism for the increasing fracture toughness in composites reinforced with SiC_w_ microfibers was explained through a detailed fracture analysis of their samples. It was revealed that upon tearing, the sample length of the composite imbued with SiC_w_ microfibers extends by up to 1 μm. This extension indicates the strong bond between the SiC_w_ fibers and the cBN matrix within the composites. Additionally, within the composite mass, the microfibers generate stresses aimed at to inhibit the expansion of existing cracks. When crack tips encounter SiC_w_ microfibers, the cracks are forced to deviate onto a more complex path, which effectively disperses energy and prevents the composite from fracturing.

Earlier research^13,14^ highlights the potential of PcBN composites enriched with oxide microfibers. Specifically, elevating the Al_2_O_3w_ and Mg_2_B_2_O_5w_ microfiber content from 5 to 15 vol% within a cBN-based composite, bonded with TaN, results in a gradual decrease in cutting tool wear. This improvement is observed even though there is a slight drop in the composite's fracture toughness (K_1C_), which can be attributed to the differing thermal expansion coefficients between the microfiber and matrix materials.

This research delves into the structural nuances, mechanical properties of bilayer composites grounded in cBN of the BL group, reinforced with both oxide and oxide-free microfibers derived from refractory compounds, alongside assessing the durability of cutting tools equiped with these materials.

## Materials and methods

Reviewing prior studies, essential characteristics have been identified for the binder material in PcBN composites. The binder should ideally possess a high modulus of elasticity and minimal differences in density and Coefficient of Thermal Expansion (CTE) compared to other composite components. Such alignment is crucial to simplify mixing processes and reduce internal stresses that could lead to cracking during the operational lifespan of cutting tools. Furthermore, the binder must exhibit chemical inertness towards materials processed during cutting and possess robust resistance to oxidation.

TiN, TiCN, and NbN compounds were used as the primary matrix component in the composites. A challenge with these components lies in their Coefficient of Thermal Expansion (CTE) mismatch with the cBN phase: TiN and TiCN exhibit a higher CTE^15^, while NbN has a lower CTE^16^. Despite these differences, their extensive research background a potential for use in PcBN cutting tools of the BL group.

The requirements for reinforcing microfibers are the same as for the matrix material, however, there is a particular focus on using microfibers of the greatest possible length and smallest diameter, since the reinforcing ability of the fibers is higher fibers with a higher length-to-diameter (*l/d*) ratio have a superior reinforcing effect. Decreasing the diameter of the fibers reduces their imperfections, significantly enhancing their strength to near-theoretical levels^17^.

This research involved analyzing two-layer composite samples: one layer reinforced with silicon carbide whiskers (SiC_w_) microfibers, and the other with alumina whiskers (Al_2_O_3w_) microfibers.

Initial samples from the PcBN BL group were produced at temperatures between 1900 and 2000 ºC and a pressure of 7.7 GPa using a high-pressure “toroid” apparatus. The materials included cBN powder with a grain size of 2–4 μm in the amount of 50–60 vol%, Al_2_O_3w_ microfibers in the amount of 20 vol% with dimensions of d = 1–2 μm and l = 50–100 μm, SiC_w_ microfibers in the amount of 5 vol%, and TiN powders with a grain size of 1–2 μm. Research^14^ indicates that adding 5 vol% of oxide-free microfibers maximizes the physical and mechanical properties of the composite. Meanwhile, incorporating up to 20 vol% of oxide microfibers enhances the composite's oxidation resistance. However, further increasing the microfiber content reduces the composite's mechanical properties.

cBN powders were finely milled to a grain size of less than 3 μm (< 3 μm) using a Fritsch 7 (Germany) planetary mill in isopropyl alcohol. Subsequently, all components of the mixture underwent ultrasonic mixing for 12–15 min to ensure thorough blending.

The compaction of the two-layer samples was performed sequentially, layer by layer. After forming the first layer, additional mixture was sprinkled on top and the sample was pressed again. Prior to sintering, the samples underwent annealing at a temperature of 600 °C for 2 h to prepare them for the sintering process.

**Figure 1:**
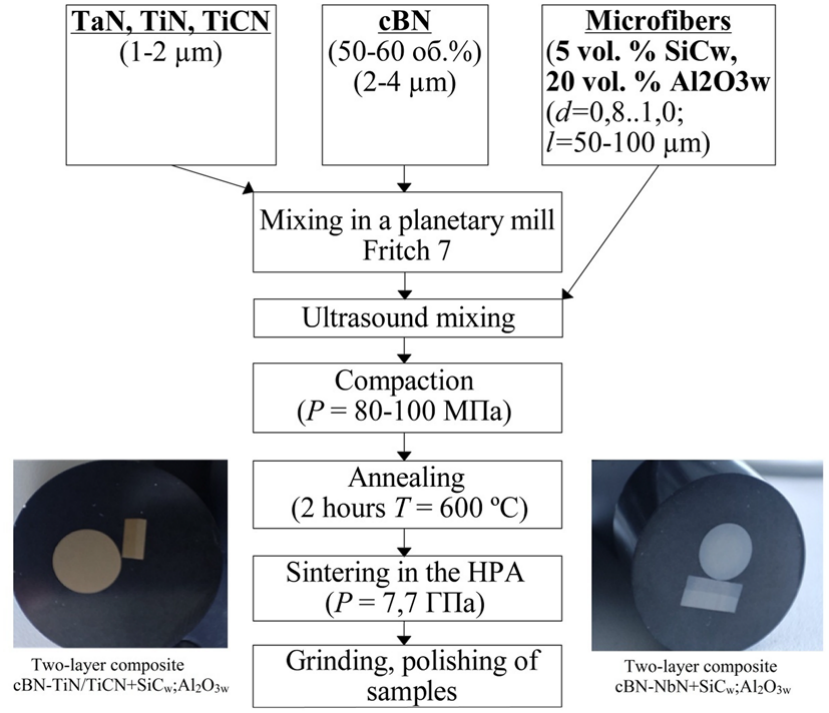
Scheme of preparation of samples of cBN cutting tools reinforced SiC_w_ and Al_2_O_3w_.

The general process of sample preparation is presented in Fig. 1.

Five samples of cutting inserts from cBN were obtained for each composition.

The microstructure of the samples was studied on a JEOLJSM-6490LV (JEOL Ltd., Japan) scanning electron microscope with an OxfordInstruments detector.

The density of the obtained samples was determined by the method of hydrostatic weighing in distilled water, using a VLR-20 (MedTech, Kyiv, Ukraine) scale (permissible weighing error of 0.025 mg). The value of ρ was calculated as the average value obtained during statistical processing of the results of 5–6 measurements. The confidence interval for the mean value was about ρ ± 0.006 g/cm^3^ with a confidence probability of 95%.

Determination of the elastic characteristics of the composites was carried out using the ultrasonic method using the thickness gauge Olympus 38 DLPlus (Japan).

The Vickers microhardness of the composites was studied on a THV-30MDX (Jinan Focus Test Instrument Co., Ltd, China) microhardness tester—the load on the indenter (a diamond pyramid with an angle at the top of 136°) did not exceed 9.8 N, the exposure time at the given load was 7 s. Measurements of the diagonals of the indenter impression after removing the load were performed using an Alicona InfiniteFocus optical microscope. Three indentations were made on each sample at the given load. To determine the microhardness of the material, the average value of the diagonal length of the indenter impression was used in the calculations. For two-layer samples, the hardness was measured separately for each layer.

The sintered blanks were subjected to finishing abrasive-abrasive processing to obtain RNGN090300T—ISO 1832:2017 (*D* = 9.52 mm, *h* = 3.18 mm) cutting plates.

Stability studies of cutting tools equipped with the created composite (layer 1—cBN 63 vol%-TiN 32 vol% + 5 vol% SiC_w_, layer 2—cBN 48 vol%-TiN 32 vol% + 20 vol% Al_2_O_3w_), were performed when turning workpieces with six evenly spaced grooves 10 mm wide and 10 mm deep from hardened HVG steel (55 HRC) with cutting speeds v = 100, 220 m/min, with feed S = 0.1 mm/rev and cutting depth t = 0.2 mm. The tool life of the cutting tool was determined by the size of the flank wear (critical value—0.3 mm), which was measured using an optical microscope directly on the machine.

For comparison, single-layer PcBN group BL composites without microfiber additives were used—cBN-TiN 40 vol%, cBN 55 vol%-TiC 45 vol%, cBN 75 vol%-TiC 25 vol%.

## Results and discussion

The study results on the density of obtained composites indicate that two-layer composites exhibit higher density levels than their single-layer counterparts (refer to Table 1). This difference likely stems from the conditions under which the samples were pre-pressed. Furthermore, among the composites, those bound with TiCN exhibit the least compactness during the sintering process. This is notably marked by the largest differences in comparison to cBN composites, both in terms of elastic modulus and coefficient of thermal expansion (CTE).

**Table 1:** Density ρ and relative density *r* of PcBN samples.

Composite	cBN-TiN		cBN-TiCN		cBN-NbN	
	ρ [g/cm^3^]	*r*	ρ [g/cm^3^]	*r*	ρ [g/cm^3^]	*r*
One-layer composite:						
Pure	4.20	98.02	4.18	88.57	5.25	–
Reinforced with Al_2_O_3w_	4.16	97.63	4.14	92.54	5.21	–
Reinforced with SiC_w_	4.12	92.16	4.12	–	5.20	97.38
Bi-layered composite	4.14	99.72	4.15	95.21	5.23	99.78

**Figure 2:**
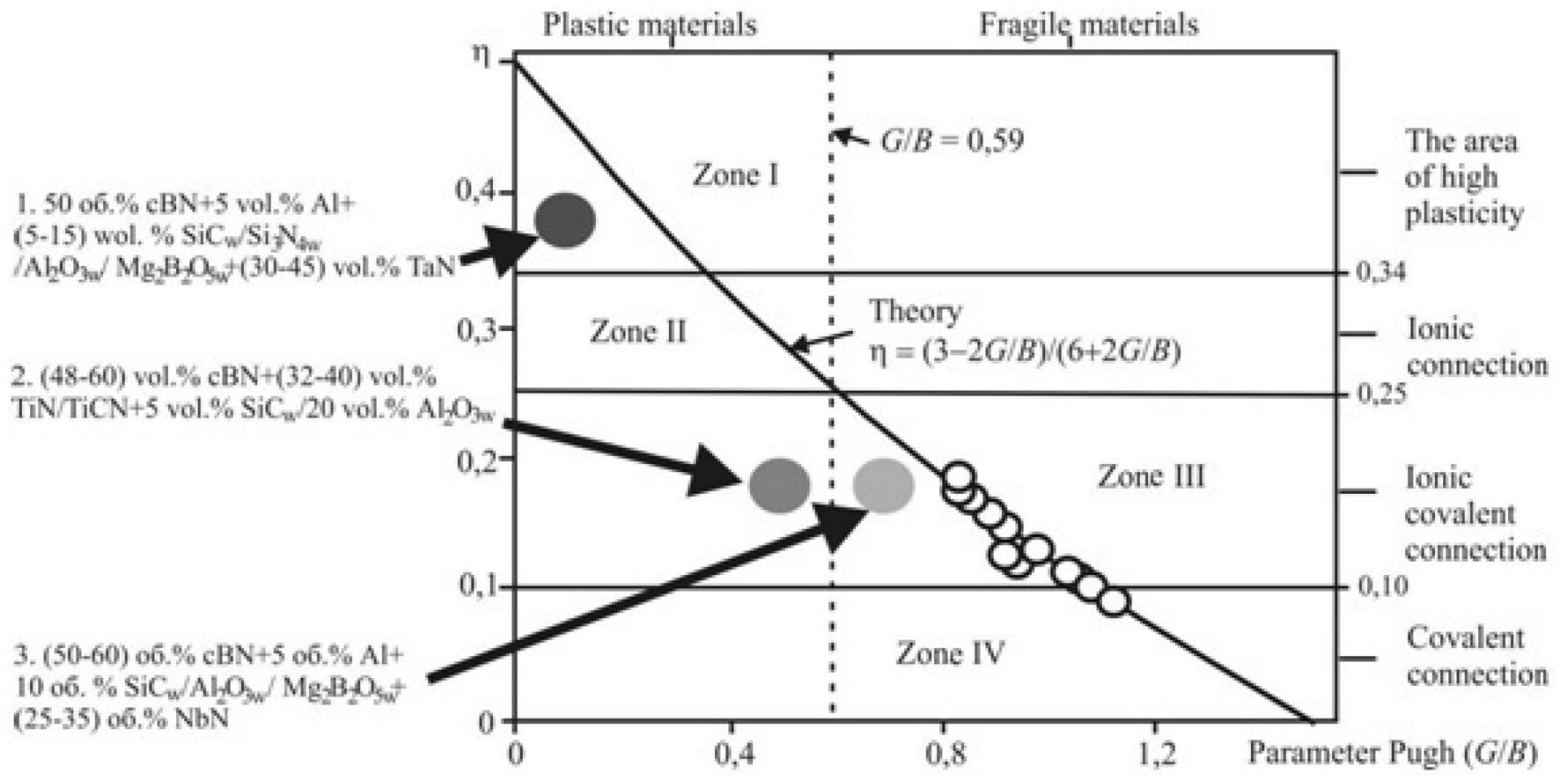
A diagram showing the dependence of Poisson's ratio values (*η*) from plasticity parameter (*G*/*B*)^19^.

The elastic properties of the obtained composites are given in Table 2. Figure 2 illustrate a diagram describing the relationship between Poisson's ratio (η) and plasticity parameter (G/B).

**Table 2:** Young's modulus *E*, Poisson's ratio µ, relations *G*/*B* of the PcBN samples.

Composite	cBN-TiN			cBN-TiCN			cBN-NbN		
	*E* [GPa]	η	*G*/*B*	*E* [GPa]	η	*G*/*B*	*E* [GPa]	η	*G*/*B*
Pure	546 ± 20	0.20 ± 0.01	0.50	675 ± 25	0.19 ± 0.01	0.51	585 ± 20	–	0.50
Reinforced Al_2_O_3w_	675 ± 25	0.18 ± 0.01	0.55	613 ± 20	0.17 ± 0.01	0.56	610 ± 20	–	0.55
Reinforced SiC_w_	755 ± 25	0.18 ± 0.01	0.55	650 ± 25	–	0.55	536 ± 20	0.20 ± 0.01	0.50

Despite the reinforcing microfibers' Young's modulus being significantly lower than that of cBN (with cBN's modulus at 900 GPa, Al_2_O_3_ ranging between 315–413 GPa, and SiC at 415 GPa), incorporating these fibers into the composite enhances the Young's modulus of the sintered PcBN. This improvement is attributed to the superior compaction achieved in samples reinforced with microfibers.

Given that the Poisson's ratio (η) for the examined PcBN materials falls between 0.17 and 0.2, this suggests they predominantly feature ionic-covalent bonds rather than metallic ones, a trait common in similar materials. It's established that when the plasticity parameter G/B is equal to or less than 0.56 (G/B ≤ 0.56), the material is considered ductile, whereas values equal to or greater than 0.57 indicate brittleness^18^.

The data presented in^19^ reveal that composites made from PcBN of the BH group are defined by a strong ionic-covalent bond and belong to brittle materials (Fig. 2). When subjected to dynamic loads, PcBN composites of the BN group exhibit a tendency towards brittle edge fracture. For cutting tools of the BL group, which are used under dynamic load conditions, it is crucial to achieve a material structural state that aligns more closely with plastic materials. This adjustment aims to minimize the occurrence of brittle edge fractures and to ensure the presence of a strong ionic-covalent or purely covalent bond.

Figure 2 illustrates that composites reinforced with both oxide and oxide-free microfibers, bonded with TaN, fall into the high plasticity zone with a significant presence of ion-metal bonds. This characteristic is deemed unfavorable for cutting tools, as such composites are prone to easy oxidation and interaction with the material being processed. In contrast, composites with TiN, TiCN, and NbN bonds predominantly exhibit strong ionic-covalent bonds. Specifically, those with NbN bonds are primarily categorized as brittle materials, making them unsuitable for cutting tools subjected to dynamic loading conditions. Furthermore, it is important to highlight that introducing aluminum (Al) into the initial compositions of composites with TaN and NbN bonds increases their brittleness. Given Al's high reactivity and its relatively low concentration in these composites, combined with the high sintering temperatures (2000 °C) used, it is reasonable to infer that almost all the added Al reacts with composite components. This reaction forms compounds characterized by high brittleness and a ratio of G/B (shear modulus to bulk modulus) ≥ 0.59. Consequently, this transformation shifts the composites from the ionic-metal zone (2) to the covalent zone (4), resulting in greater brittleness.

**Figure 3:**
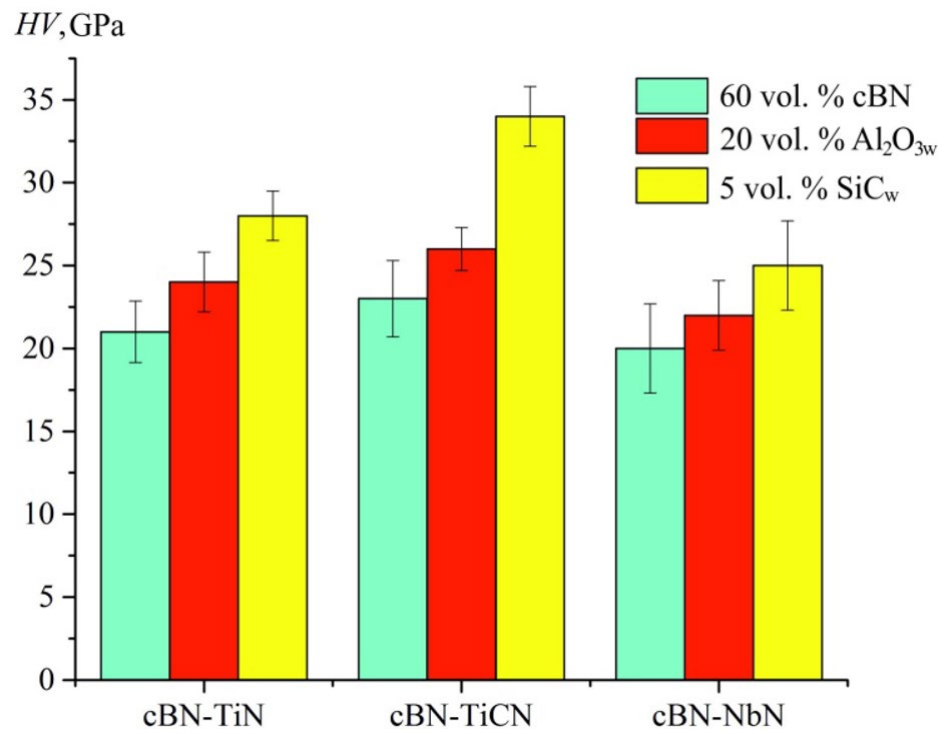
Hardness of the obtained samples of composites.

The hardness of the obtained composites used in the research is presented in Fig. 3.

The incorporation of microfibers into the mix for two-layer polycrystalline cubic boron nitride (PcBN) composites enhances their hardness. This improvement is attributed to the effect of double compaction, as well as the microfibers' ability to slow down the spread of dislocations within the elastic–plastic zone during indentation tests. Among the composites, those reinforced with silicon carbide whiskers (SiC_w_) exhibit the highest hardness levels. This superior hardness is linked to the greater values of Young's modulus and inherent hardness of the SiC_w_ microfibers compared to other types used.

**Figure 4:**
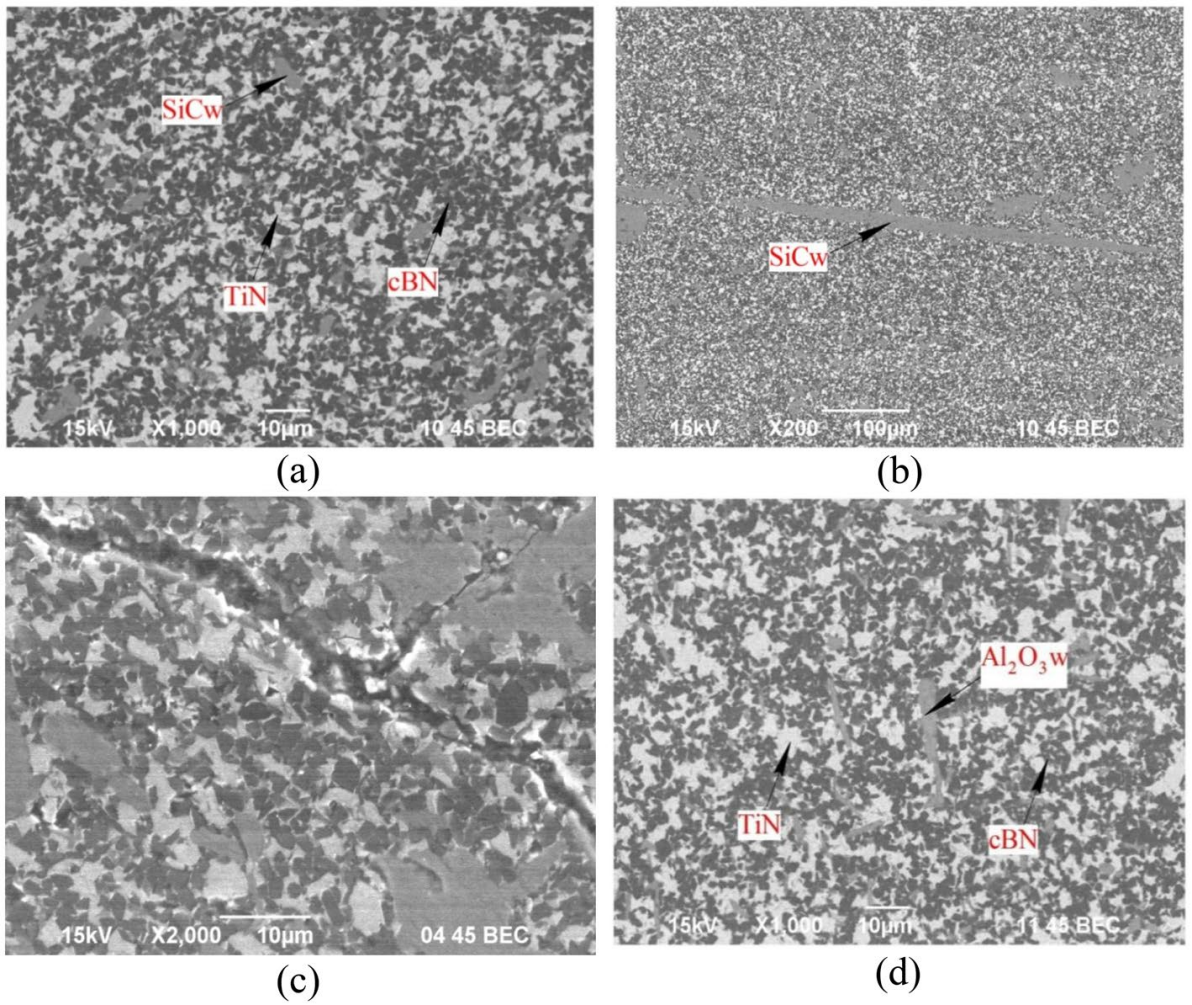
SEM image of the structure of a cBN-based composite with a TiN bond reinforced with SiC microfibers (**a**, **b**), a crack on the surface of the composite (**c**) reinforced with Al_2_O_3w_ microfibers.

Microstructural analysis of a two-layer composite featuring a TiN-based bond revealed a uniform phase distribution: dark regions correspond to the cubic cBN phase, while gray areas are indicative of the bonding phase (Fig. 4).

SiC_w_ microfibers within the composite are mainly found as chopped fragments ranging from 5 to 10 μm in length (Fig. 4a). However, in certain areas, these microfibers extend up to 300 μm (Fig. 4b). The surface of the samples displays a network of microcracks (Fig. 4c), a feature attributed to the stress–strain state of the composite. This condition results from the disparity in the CTE among cBN, TiN, and SiC_w_ microfibers. The microfibers undergo destruction during the charge mixing process, which compromises their primary role of inhibiting crack formation.

Al_2_O_3w_ microfibers are uniformly dispersed throughout the composite structure (Fig. 4d), with sizes ranging from 10 to 20 μm. This uniformity suggests that the microfibers remain largely intact, without significant mechanical degradation, during the charge formation and sintering processes of the composite. Additionally, the surface of the resultant samples shows no significant cracks, indicating the microfibers' effectiveness in maintaining the structural integrity of the composite.

**Figure 5:**
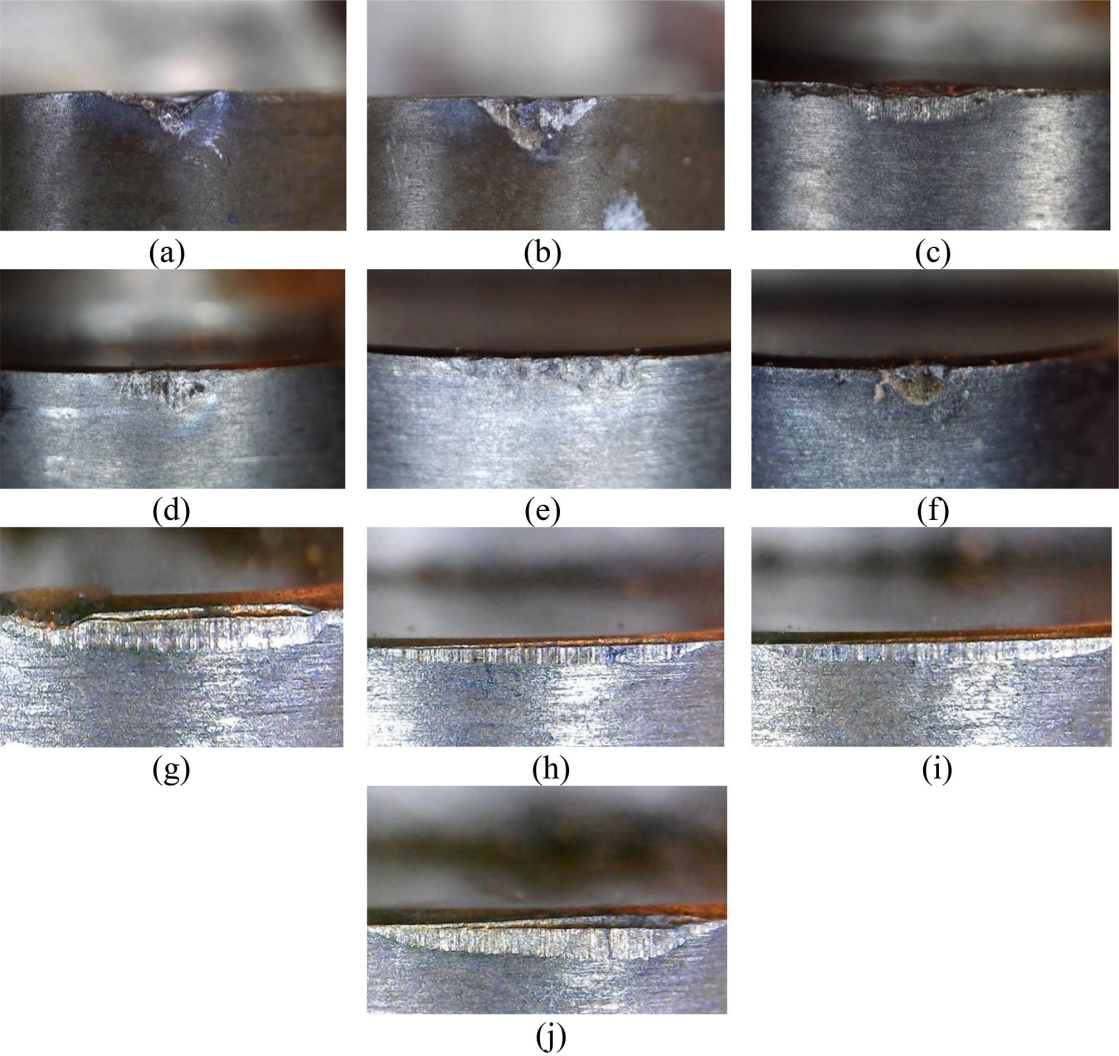
Flang wear of the PcBN cutting tools after the impact precision of hardened steel HVH (**a**, **c**, **e**, **g**, **i** 100 m/min; **b**, **d**, **f**, **h**, **j** 220 m/min): (**a**–**f**) one-layer composites (**a**, **b** cBN-TiN 40 vol%; **c**, **d** cBN 55 vol%-TiC 45 vol%; **e**, **f** cBN 75 oб.%-TiC 25 oб.%); **g**–**j** two-layer composites (**g**, **h** cBN 48 vol%-TiN/TiCN 32 vol% + 20 vol% Al_2_O_3w_; **i**, **j** layer of cBN 63 vol%-TiN/TiCN 32 vol% + 5 vol% SiC_w_).

Research on the performance of cutting tools equipped with the newly developed composites indicates that, when subjected to shock loading conditions and operating at cutting speeds of up to 100 m/min, there is noticeable mechanical wear on the cutting edge. Moreover, abrasive interactions with the workpiece material at the contact surfaces are observed, as illustrated in Fig. 5.

With the increase in cutting speed to 220 m/min, the resilience of PcBN cutting tools under impact loads experiences a marginal enhancement. This improvement is associated with the softening of the material being processed, which is induced by the high temperatures during the cutting process (Fig. 5). At these elevated speeds, a principal factor in tool wear is the chemical interaction between the components of the cutting tool's composite and the workpiece material. This process encompasses both the oxidation of the cutting tool's composite components and the diffusion of elements from the composite into the material being processed.

The data obtained indicate that when processing with a shock load using a cutting tool made of a cBN-TiN (40 vol%) PcBN composition, brittle chipping of the cutting edge occurs on both the front and back surfaces (Fig. 5a) just 30 s after starting operation. This indicates low mechanical strength of the composite. In contrast, employing a cutting tool with a TiC bond, known for its higher Young's modulus and hardness values compared to TiN, showed predictably improved outcomes (Fig. 5c). Nonetheless, the type of cutting edge failure, characterized by volumetric cracking, also points to inadequate mechanical properties of this composite. The onset of cutting edge destruction with the TiC-bonded cutting tool occurs after 4 min, indicating a somewhat increased but still limited durability.

Elevating the concentration of cBN grains within the composite to 75 vol%, in combination with utilizing a TiC binder, allowed to enhance the cutting tool's stability to up to 6 min. It is important to highlight that for this specific cutting tool configuration, the contact area on the back surface (Fig. 5e) is predominantly shaped by volumetric eruptions from the composite's components. This phenomenon generates an effect of serration on the cutting edge, which, in turn, accelerates the cutting tool's abrasive wear rate. The emergence of an irregularly worn cutting edge can significantly degrade the surface finish of the workpiece processed.

The lifespan of a cutting tool fitted with a newly formulated composite, consisting of 48 vol% cBN, 32 vol% TiN, and 20 vol% Al_2_O_3w_ microfibers, under comparable operational conditions, extends to 7 min. The primary cutting tool wear is caused mainly by abrasive interaction on the contact areas, as evidenced by the grooves on the back surface (Fig. 5c). Remarkably, there are virtually no chips on the cutting edge, which essentially retains its straightness throughout the operation.

Integrating SiC_w_ microfibers with a TiN binder enhances the mechanical properties of PcBN composites. The wear pattern observed in cutting tools utilizing this composite primarily stems from abrasive interactions with the material being processed. This leads to the creation of a hole on the front surface, along with mechanical degradation and chipping of the cutting edge. Although the SiC_w_ microfiber-reinforced composite exhibits marginally superior mechanical properties and demonstrates a similar width of the wear bevel on the cutting tool's rear surface as that of the cutting tool reinforced with Al_2_O_3w_ microfibers, it experiences partial destruction on both the front and rear surfaces. This damage is attributed to the presence of cracks and a considerable degree of internal stresses within the composite.

Study^20^ demonstrates that increasing the cutting speed to 200–300 m/min, especially under shock load conditions, can reduce the cutting force by up to 1.5 times. This reduction is attributed to the decreased deformation of the material being processed, resulting from its softening due to the high temperatures generated in the cutting zone, which can reach 1100–1200 °C. In the context of high-speed processing, involving shock loads, the material's mechanical properties—such as crack resistance, hardness, and Young's modulus—are crucial. However, equally important is the material's capacity to withstand chemical effects at the cutting tool's contact areas during processing. This includes resisting oxidation of the cutting tool material and preventing the diffusion of its components into the material being processed, phenomena that are particularly activated under high-temperature conditions.

During high-speed processing with shock loads on HVH steel with a cutting tool made of PcBN cBN-TiN mixture at 40 rev.%; the formation of microchips, on both the front and back surfaces of the cutting tool is noted, alongside a high intensity of wear observable as early as 60 s into operation (Fig. 5b).

In the cutting tool's contact area, characteristic traces of high-temperature effects in the cutting zone are evident, such as a “metallic” colored zone. This may indicate adhesion of the material processed to the cutting tool. Probably, this phenomenon, coupled with shock loads, causes the formation of chips in the composite material (Fig. 5d).

Elevating the proportion of BN grains to 75 vol% within the composite's composition has led to reduced chamfer wear of the cutting tool, attributed to the enhancement of the PcBN's mechanical properties (Fig. 5f). However, after 4 min of cutting under impact conditions, the tool's cutting edge experiences chipping, making further usage impossible. The wear mechanism for cutting tools employing this composite is linked to mechanical destruction, temperature effects, and abrasive interaction with the material processed.

When employing a cutting tool equipped with a two-layer composite reinforced with SiC_w_ and Al_2_O_3w_ microfibers it was assumed that during high-speed processing, the layer containing SiCw microfibers will act as a rigid foundation, bolstering the strength of the working layer reinforced with Al_2_O_3w_ fibers. In turn, the Al_2_O_3w_ fibers will contribute to reducing the oxidation intensity of the composite when exposed to high temperatures > 822 °C.

Utilizing a two-layer cutting tool made of a composite reinforced with Al_2_O_3w_ microfibers (Fig. 5h) results in a durability of 7 min. This configuration showed the best stability characteristics during high-speed processing under shock loads.

The inclusion of just 20% Al_2_O_3w_ microfibers has been observed to diminish cutting tool wear associated with chemical reactions in the cutting zone. The crystallized liquid phase traces on the cutting tool's contact areas is linked to the contact-reactive melting mechanism—namely, the melting of products chemical interaction of cutting tool material and materials being processed^21^. The primary failure mechanism of this cutting tool is identified as the abrasive wear of the contact areas.

It's important to highlight that oxide fibers, due to their chemical properties, are inert and do not react with either oxygen or iron-based alloys. This characteristic significantly contributes to their utility in environments where chemical stability under high temperatures or reactive conditions is crucial.

The results demonstrate that for the cutting tool of PcBN reinforced with SiC_w_ microfibers, the predominant wear mechanism is the chemical interaction with the material being processed. This involves the oxidation of the cutting tool material, the interaction between the materials in contact, and the diffusion of elements from the PcBN into the material being processed. Evidence of this mechanism includes the presence of hole wear on the front surface as well as lowering of the tool's cutting edge (Fig. 5j).

The likelihood of chemical interactions between SiCw fibers, the components of the material being processed, and air oxygen is evident. Model studies on the interaction between SiC and Fe, detailed in^22^, demonstrate that silicon can dissolve in iron at a temperature of 1223 K. Furthermore, the oxidation of SiCw microfibers, leading to the release of free carbon, begins at a temperature of 1200 °C^23^. This process also results in the formation of an amorphous SiO_2_ layer on the surface, which is separated by a thin layer of SiNxOy or oxynitride Si_2_N_2_O. Such chemical transformations weaken the microfibers, diminishing their ability to effectively reinforce the composite material.

**Figure 6:**
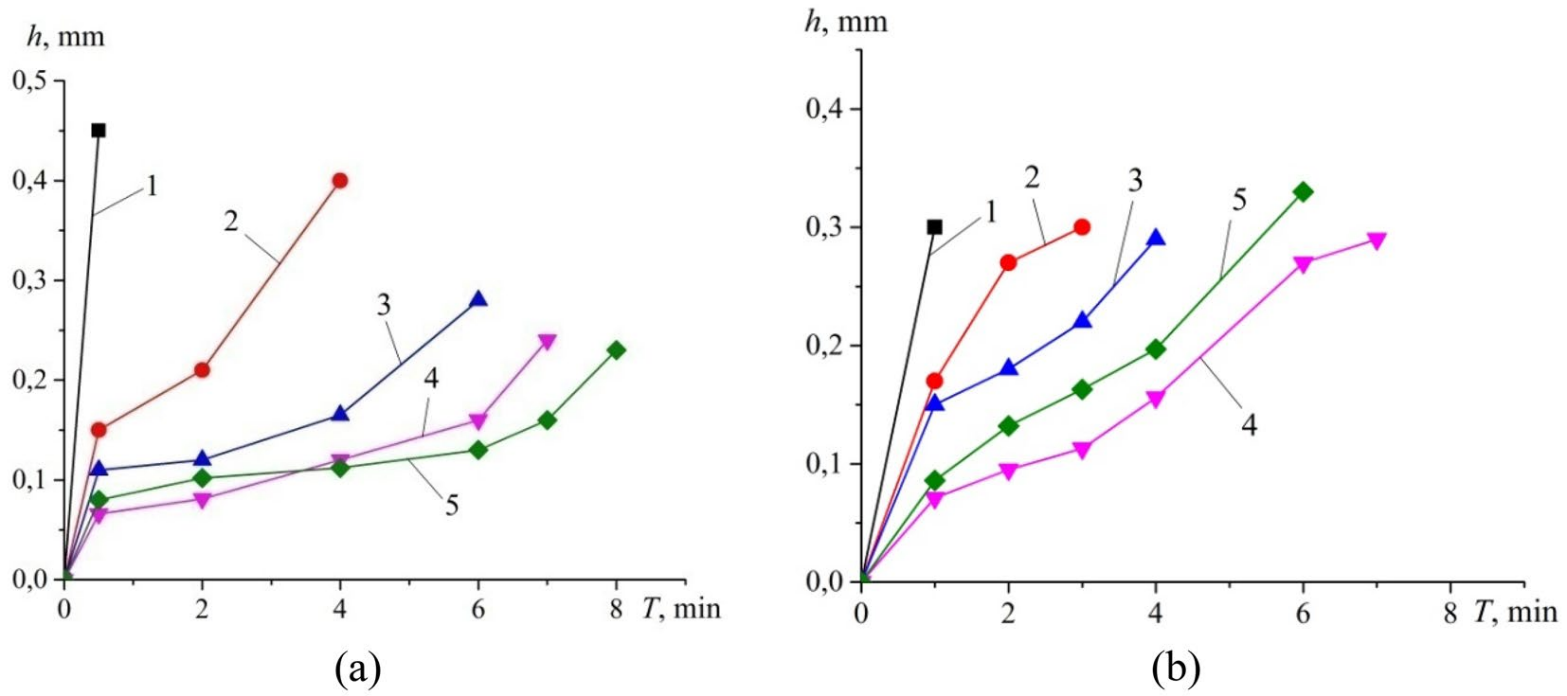
Kinetics of flank wear for a cutting speed of 100 m/min (**a**) and 200 m/min (**b**): 1–3—one-layer composites (1—cBN-TiN 40 vol%; 2—cBN 55 vol%-TiC 45 oб.%; 3—cBN 75 vol%-TiC 25 vol%); 4, 5—two-layered composites (4—layer of cBN 48 vol%-TiN/TiCN 32 vol% + 20 vol% Al_2_O_3w_; 5—layer of cBN 63 vol%-TiN/TiCN 32 vol% + 5 vol% SiC_w_.

The generalized results of determining the duarability of cutting tools equipped with the created composites for different cutting speeds are presented in Fig. 6.

## Conclusions

1. It has been established that the density of two-layer composites reinforced with microfibers SiCw and Al_2_O_3w_ is higher than that of single-layer composites, which is attributed to the pre-pressing of samples, namely, after forming the first layer, the mixture was topped up and re-pressed.
2. Requirements for composites reinforced with SiC_w_ and Al_2_O_3w_ microfibers were formulated based on a diagram illustrating the relationship between Poisson's ratio (η) and the plasticity parameter (G/B). According to this diagram, for tools belonging to the BL group used in dynamic load machining, it is imperative to maintain the material in a structural state within the plastic zone to facilitate robust ion-covalent or covalent bonding.
3. Bonds based on TiN, TiCN, NbN, TaN with the addition of Al were analyzed. It was found that TiN and TiCN bonds exhibit the most optimal properties necessary for creating BL group composites for processing under dynamic load conditions due to the formation of strong ion-covalent bonds.
4. The Young's modulus, Poisson's ratio, and hardness (HV) of the obtained two-layer composites reinforced with SiCw and Al2O3w microfibers with TiCN and NbN bonds were determined. It was found that the addition of microfibers to the composite resulted in increased hardness and Young's modulus of sintered PcBN due to enhanced densification of reinforced samples. Additionally, microfibers aided in reducing the speed of dislocation propagation in the elastic–plastic zone during indentation.
5. It was shown that when processing hardened steel with impact loads at cutting speeds up to 100 m/min, the highest tool life is inherent for the cutting tool with the two-layer composite and a working layer reinforced with SiC_w_.
6. In conditions where the cutting speed reaches 200 m/min, the optimal performance is observed for two-layer cutting tools reinforced with Al_2_O_3w_ microfibers. This is likely attributed to a reduction in the intensity of the chemical reaction between the cutting tool material and the workpiece, while the layer reinforced with SiCw microfibers provides a rigid substrate to prevent mechanical tool failure.

## Data Availability

The datasets used and/or analysed during the current study available from the corresponding author upon reasonable request.
